# Managing public-private partnerships in public health nutrition requires ‘deep professionalism’: Editorial comment on ‘Managing engagement among public, private and civil society actors participating in NewTools: a research partnership on food profiling’

**DOI:** 10.1017/S1368980025100608

**Published:** 2025-07-04

**Authors:** Luc Louis Hagenaars, Laura Anne Schmidt

**Affiliations:** 1 Amsterdam UMC Location University of Amsterdam, Department of Public and Occupational Health, Meibergdreef 9, Amsterdam, The Netherlands; 2 University of California San Francisco, School of Medicine, 490 Illinois Street, San Francisco, CA, USA

Conflicts of interest in nutrition science and policy are major obstacles to promoting public health nutrition^(1)^ and, unfortunately, are more often the rule than the exception^(2)^. In the 1960s, for example, the sugar industry sponsored prominent scientists at Harvard to publish reviews that contributed to focusing scientific attention on fats and cholesterol as primary dietary culprits, with sugar attracting less attention^(3)^. In the 2010s, Coca-Cola funded an international nutrition research network to promote claims that sedentary behaviors were important drivers of obesity, thereby shifting attention away from soda and ultraprocessed food consumption^(4)^.

These industry tactics often operate to steer research agendas rather than to outright deny opposing evidence, making them hard to refute. This helps explain the ‘nutritionism’ paradigm that led to a focus on the health effects of single foods or single nutrients^(5)^. ‘Nutritionism’ has fueled ‘food fads’ that distracted from more important research on whole diets^(6)^. Ample studies show that food industry efforts have sought to control the nutrition science narrative by creating confusion and doubt; delay and derail evidence-based policy measures such as soda taxes, government subsidy reforms, front-of-package warning labels, and health-promoting procurement policies^(7–9)^.

It is in this context that the Norwegian NewTools Consortium^(10)^ sought to develop a framework for cross-sector partnerships in collaborative food systems modeling, bringing together academic, governmental, civil society, and domestic food industry stakeholders^(10)^. Their framework adapts the World Health Organisation’s (WHO) *Framework for Engagement with Non-State Actors* ^(11)^, pertaining to multinational corporations, with literature and internal discussions among consortium members, resulting in four key components: defining overarching principles for collaboration, describing roles and responsibilities of the partners involved, establishing procedures to ensure involvement and transparency, and identifying and mediating potential conflict areas. Through such a voluntary collaboration, the NewTools Consortium seeks to navigate the competing interests, conflicts of interest, and other tensions between stakeholders to develop food profiling models for dietary quality and environmental and societal impact in Norway.

A noteworthy element of the NewTools framework is its explicit rejection of consensus as a precondition for decision making in food systems modeling. The framework assumes that there will be disagreement among stakeholders, that not all input from all partners will be considered, and ultimately, leaves modeling decisions up to researchers who rely on scientific evidence and scientific rationales. While a helpful safeguard in theory, it remains to be seen how such principles will play out in practice. Will cross-sector partnerships remain intact after researchers make decisions that oppose food industry interests? Or in practice, will these partnerships simply still strive for consensus?

The NewTools framework tries to create a structured way for government and civil society stakeholders to engage with industry without allowing industry interests and agendas to drive decision-making. The literature on scientific conflicts of interest, however, suggests that industry influence often operates in subtle, unconscious ways that are difficult to manage and control^(12)^. Take the case of physicians who change their prescription practices after receiving small gifts and meals from pharmaceutical companies, all while denying that any influence has occurred^(13)^. In a similar vein, nutrition researchers often claim in journal disclosures that their industry funders have had no influence on their research. Meanwhile, meta-analyses objectively document large and consistent effects for industry funding bias across the nutrition science literature^(14)^. Research shows that industry funders often impact research by shaping the contexts in which scientists frame their research questions^(13)^. These subtle, yet pervasive, forms of bias may similarly impact cross-sector partnerships in food systems modeling, such as the NewTools Consortium.

According to the psychologist Sunitah Sah, to effectively manage conflicts of interest, we need to combine policies with developing and fostering ‘deep professionalism’: a ‘consistent practice of behaviors, and full understanding of the limits of self-regulation via reflection, moral development, and intellectual humility’^(15)^. For public health nutrition researchers, this means not only implementing structures such as those outlined in the NewTools framework, but also reducing conflicts of interest as far as possible, for example by prohibiting acceptance of even small gifts from interested parties. In addition, public health nutrition researchers should develop deep professionalism with educational activities and practices that enhance understanding of the psychological dynamics underpinning conflicts of interest. These should emphasize intellectual humility and the consistent practice of behaviors embodying this understanding, especially among leadership. Concrete expressions of this ethos might include refusing all gifts, even when institutional policy permits small gifts under $100; disclosing in conflicts of interest statements that ‘It is unknown how much I’ve been influenced’; and making a habit of seeking out evidence that challenges industry positions after interactions with an industry actor.

It remains to be seen if the NewTools framework provides a sophisticated enough structure for protecting nutrition science and policy from private sector interests, beyond what is already outlined in the WHO *Framework for Engagement with Non-State Actors* ^(11)^. Since industry influence is likely to be subtle and hard to monitor, successful implementation and evaluation of the NewTools framework may require promoting ‘deep professionalism’ across executive and leadership levels.
